# Integrative Analysis of ATAC-Seq and RNA-Seq through Machine Learning Identifies 10 Signature Genes for Breast Cancer Intrinsic Subtypes

**DOI:** 10.3390/biology13100799

**Published:** 2024-10-07

**Authors:** Jeong-Woon Park, Je-Keun Rhee

**Affiliations:** Department of Bioinformatics & Life Science, Soongsil University, Seoul 06987, Republic of Korea; wjddns037@gmail.com

**Keywords:** breast cancer, RNA-seq, ATAC-seq, integrative analysis, machine learning algorithms, breast cancer intrinsic subtype

## Abstract

**Simple Summary:**

Breast cancer is categorized into four main intrinsic subtypes, and distinguishing between these subtypes is crucial for providing personalized treatment to patients. However, systematic analyses exploring the connections between gene expression and chromatin accessibility using bulk RNA-seq and ATAC-seq data, coupled with machine learning algorithms, are lacking. In this study, we develop a classification model based on the integrative analysis of RNA-seq transcriptome and ATAC-seq epigenetic information. We identify 10 signature genes associated with these intrinsic subtypes, which are predominantly linked to immune responses, hormone signaling, cancer progression, and cellular proliferation.

**Abstract:**

Breast cancer is a heterogeneous disease composed of various biologically distinct subtypes, each characterized by unique molecular features. Its formation and progression involve a complex, multistep process that includes the accumulation of numerous genetic and epigenetic alterations. Although integrating RNA-seq transcriptome data with ATAC-seq epigenetic information provides a more comprehensive understanding of gene regulation and its impact across different conditions, no classification model has yet been developed for breast cancer intrinsic subtypes based on such integrative analyses. In this study, we employed machine learning algorithms to predict intrinsic subtypes through the integrative analysis of ATAC-seq and RNA-seq data. We identified 10 signature genes (*CDH3*, *ERBB2*, *TYMS*, *GREB1*, *OSR1*, *MYBL2*, *FAM83D*, *ESR1*, *FOXC1*, and *NAT1*) using recursive feature elimination with cross-validation (RFECV) and a support vector machine (SVM) based on SHAP (SHapley Additive exPlanations) feature importance. Furthermore, we found that these genes were primarily associated with immune responses, hormone signaling, cancer progression, and cellular proliferation.

## 1. Introduction

According to GLOBOCAN 2020 data, breast cancer is the most prevalent cancer among women and the fifth leading cause of cancer-related deaths [1]. It exhibits high heterogeneity at both morphological and molecular levels. Breast cancer is primarily categorized into four intrinsic subtypes—Luminal A, Luminal B, Basal-like, and HER2-enriched—based on the expression profiles of 50 genes, commonly known as PAM50 [2]. While the PAM50 gene signature is valuable for classifying subtypes and guiding treatment decisions, it has some limitations, such as its reliance on numerous genes which increases both the costs and complexity of analyses in research and clinical settings [3].

In addition to the molecular subtyping based on the gene expression, extensive research has aimed to identify risk factors for breast cancer, with genome-wide association studies (GWASs) highlighting common genetic variations associated with the disease [4,5]. However, these genetic factors only partially account for the heritable risk. Emerging evidence suggests that epigenetic changes could play a critical role in the development and progression of breast cancer, influenced by environmental and dietary factors [6,7,8]. This underscores the need for a comprehensive understanding of epigenetic factors in the breast cancer.

Recent studies have advanced our understanding of epigenetic mechanisms underlying breast cancer subtypes. For instance, Hector et al. demonstrated that subtype-specific enhancers influence gene expression patterns in breast cancer using RNA-seq, GRO-seq, and ChIP-seq [9]. Similarly, Soledad et al. identified molecular differences related to PAM50 gene signature that may lead to functional variations [10]. They selected 13 coding transcripts and two microRNAs (MiR-21 and miR-10b) for the four breast cancer subtypes by integrating gene expression, DNA methylation, and microRNA expression data.

Another important epigenetic factor is chromatin accessibility, which plays a vital role in gene regulation by allowing access to transcription factors and other regulatory proteins [11]. ATAC-seq (assay for transposase accessible chromatin with sequencing) is a powerful technique that identifies the open chromatin regions, providing insights into gene expression regulation and its variability across cell types and conditions [12]. Yuexin et al. highlighted subtype-specific promoter regions, such as *FOXC1* and *ESR1*, which differ between basal and non-basal breast cancer [13].

However, to our knowledge, no systematic analysis has yet explored the connections between gene expression and chromatin accessibility using RNA-seq and ATAC-seq, particularly coupled with machine learning algorithms, to differentiate between breast cancer subtypes. Although several studies have combined machine learning algorithms with epigenetic information, most of these primarily used DNA methylation profiling datasets [14,15]. Integrating the RNA-seq transcriptome data with the ATAC-seq epigenetic information yields a more comprehensive understanding of gene regulation and its impact in breast cancer [16].

Here, we utilized bulk RNA-seq and ATAC-seq data to explore the association between gene expression and chromatin accessibility at promoter regions in breast cancer patients. The primary objectives and contributions of the present research are (1) to develop a classification model to predict breast cancer intrinsic subtypes based on gene expression levels obtained from the integrated RNA-seq and ATAC-seq dataset, (2) to identify signature genes for the molecular classification of the breast cancer intrinsic subtypes, and (3) to search for potential transcription factor binding sites associated with these marker genes. Overall, our study demonstrates that combining RNA-seq and ATAC-seq data with machine learning algorithms can enhance our understanding of chromatin accessibility in breast cancer intrinsic subtypes and provide valuable insights into the molecular mechanisms driving the progression of these subtypes.

## 2. Materials and Methods

### 2.1. Data Preparation and Integration

In this study, four gene expression datasets—GDC TCGA-BRCA, GSE96058, GSE81538, and GSE135298—were obtained from the UCSC Xena browser (https://xenabrowser.net/, accessed on 15 August 2024) and the Gene Expression Omnibus (GEO) database (https://www.ncbi.nlm.nih.gov/geo/, accessed on 15 August 2024) [17,18,19]. The gene expression data were transformed to log_2_(TPM + 1). These datasets were generated by different institutions. Genes overlapping with gene symbols from GENCODE version 22 and GEO datasets were selected, excluding genes on sex chromosomes and mitochondrial chromosomes. For genes with multiple Ensembl IDs, the genes with the longest gene length were used.

ATAC-seq peak data for GDC TCGA-BRCA were also obtained from the UCSC Xena browser (https://xenabrowser.net/, accessed on 15 August 2024). The data are represented as log_2_(count + 5)PM-qn, where raw counts were adjusted by adding 5, converted to counts per million (CPM), and then transformed using log2 and quantile normalization.

Clinical information from GDC TCGA-BRCA patients was extracted using the R package TCGAbiolinks version 2.32.0 (accessed on 15 August 2024) [20]. Patients from the primary vial (e.g., −01A) were selected, excluding individuals with technical replicates and those classified under the normal-like subtype. Similarly, clinical data from GEO gene expression datasets were processed using the R package GEOquery version 2.72.0 [21].

### 2.2. Integrative Analysis between RNA-Seq and ATAC-Seq in GDC TCGA-BRCA

Accessible peaks from preprocessed TCGA-BRCA peak calling data were annotated using the R package ChIPseeker version 1.40.0 to search the nearest transcription start site (TSS) [22,23]. Promoter regions were defined as areas within 2 kb upstream and downstream of the TSS, and peaks located in promoter regions were used for the further analysis. Gene annotation was performed using the R package org.Hs.eg.db version 3.19.1. Initially, peaks on sex chromosomes and mitochondrial chromosomes were excluded for consistency. When multiple peaks were assigned to the same gene, the peak with the highest normalized score was chosen.

RNA-seq and ATAC-seq data from 68 matched GDC TCGA-BRCA patients were used for the integrative analysis. Genes and peaks with low variability, specifically a median gene expression ≤ 1 and median peak signal ≤ 1, were excluded. A Spearman correlation analysis was conducted to examine the relationship between gene expression and chromatin accessibility at promoter regions in the matched patients. Correlations with a coefficient ≥ 0.65 and a *p*-value < 0.01 were considered significant.

### 2.3. Data Preprocessing and Feature Selection

Gene expression and clinical data from the GSE96058 dataset were utilized to develop a classifier for breast cancer intrinsic subtypes. The dataset included genes that exhibited significant correlations between promoter accessibility and gene expression, as identified from matched GDC TCGA-BRCA patients. The data were generated using both the Illumina HiSeq 2000 and Illumina NextSeq 500 sequencers. For model development and evaluation, patients with a GPL1154 platform ID (from the Illumina HiSeq 2000) were used, while patients with a GPL18573 platform ID (from the Illumina NextSeq 500) were used for external validation. The preprocessed GSE96058 dataset was divided into training data (70%) and test data (30%). Subsequently, data normalization was applied separately to the training and testing sets by calculating the Z-score [24].

Feature selection was performed using recursive feature elimination with cross-validation (RFECV), with a support vector machine (SVM) as the estimator and based on SHAP (SHapley Additive exPlanations) feature importance [25]. This approach was employed to identify optimal genes, aiming to enhance both accuracy and computational efficiency. This process used stratified 10-fold cross-validation, repeated ten times, to enhance the robustness of the model performance estimates. All the data preprocessing and feature selection experiments were conducted using the Python library probatus version 3.1.0 and scikit-learn version 1.5.1.

### 2.4. Construction and Evaluation of Machine Learning Algorithms

Machine learning algorithms were developed using the selected feature sets. Six algorithms were employed: SVM (support vector machine), LR (logistic regression), NB (naïve Bayes), AdaBoost (adaptive boosting based on decision trees), and MLP (multilayer perceptron).

Before constructing the classification algorithms, the distribution of breast cancer subtypes was assessed, revealing an imbalance that could bias the models. We applied a clustering-based under-sampling technique, replacing majority class samples with centroids identified using the k-means algorithm [26]. The performance of models trained on the under-sampled data was compared with those trained on the original data. This comparison aimed at evaluating improvements in classification accuracy, ensuring robust performance estimation and minimizing prediction errors.

To visualize the gene expression profile of the training data, the uniform manifold approximation and projection (UMAP) method was employed. Optimal hyperparameter values for each model were determined through grid search with stratified 10-fold cross-validation, repeated 10 times. The hyperparameter settings for this process are detailed in Table S1. Model performance was evaluated using several metrics: accuracy, specificity, sensitivity, F1 score, and AUROC (area under the receiver operating characteristic curve). For multiclass classification, all metrics were evaluated using the macro-average method for a comprehensive assessment across all classes, except for accuracy.

All the experiments using the machine learning were conducted using the Python libraries imbalanced-learn version 0.12.3, umap-learn version 0.5.6, scikit-learn version 1.5.1, and Yellowbrick version 1.5.

### 2.5. Validation with Independent Gene Expression Datasets

To assess the generalizability and robustness of the model, we used external validation datasets—GSE81538, GSE135298, and GSE96058—that were not involved in the model construction phase. These datasets also normalized by calculating Z-scores at the machine learning model construction.

Additionally, further validation was performed using the GDC TCGA-BRCA dataset, which was not included in the initial correlation analysis. This dataset was split into a training set (70%) and a test set (30%), and underwent Z-score normalization. The model was then retrained on the GDC TCGA-BRCA training set with genes selected through feature selection, and its prediction performance was subsequently evaluated.

### 2.6. GO and KEGG Enrichment Analysis

To elucidate the biological significance of the selected genes identified through feature selection, Gene Ontology (GO) and Kyoto Encyclopedia of Genes and Genomes (KEGG) were used for enrichment analyses using the R package clusterProfiler version 4.12.1 [27,28,29]. Biological process terms were considered enriched if they had a *q*-value < 0.05, which represents the adjusted *p*-value using the Benjamini–Hochberg method for controlling false discovery rates [30,31]. KEGG pathways were deemed enriched with a *q*-value < 0.1. Visualization of the enrichment results was carried out using the R package enrichplot version 1.24.2.

### 2.7. Motif Discovery Analysis

To identify peak locations of genes selected through feature selection within promoter regions, ATAC-seq data from the GDC TCGA-BRCA cohort were utilized. DNA sequences corresponding to these peak locations from the masked human reference genome version 38 (https://genome.ucsc.edu/, accessed on 15 August 2024) were extracted using bedtools version 2.31.1 [32]. Subsequently, MEME-ChIP (version 5.5.5) was employed to analyze these sequences and identify both novel DNA-binding motifs and known transcription factor binding sites associated with the selected genes. Motifs for analysis were sourced from the JASPAR 2024 database (https://jaspar.elixir.no/, accessed on 15 August 2024) [33,34]. Motifs with an E-value < 0.01 were considered as significantly enriched, reflecting a low probability of the alignment occurring by chance. This approach integrates genomic sequence analysis with motif discovery to uncover potential regulatory elements influencing the selected genes.

## 3. Results

### 3.1. Integrative Analysis of Gene Expression and Promoter Accessibility in Breast Cancer Patients

We utilized RNA-seq and ATAC-seq data from the GDC TCGA-BRCA cohort, which included 17,217 gene expression profiles from 1021 breast cancer patients and 215,920 chromatin accessibility profiles from 70 of these patients. Promoter regions were defined as areas within 2 kb upstream and downstream of the transcription start sites (TSSs).

First, we examined the genomic distribution of the 215,920 accessible peaks identified from the ATAC-seq data. As shown in Figure S1A, the largest proportion of these peaks (33.43%) were in promoter regions (≤1 kb and 1–2 kb). The second most frequent region was distal intergenic regions, accounting for 22.78%. Figure S1B illustrates the distribution of distances from the peaks to the nearest genes, with peaks most frequently found in the 10–100 kb region, followed by the 0–1 kb region.

**Figure 1:**
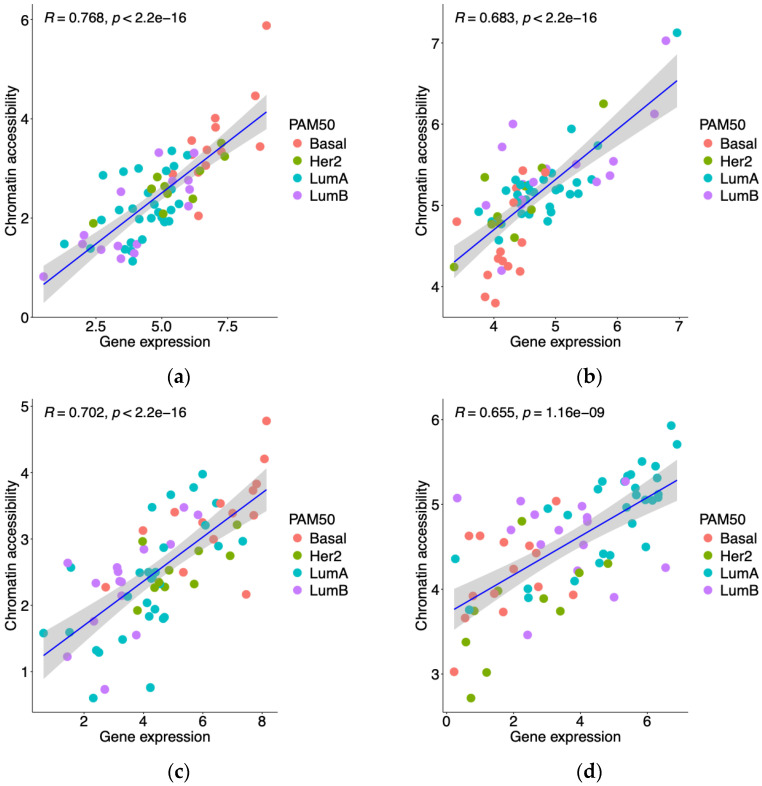

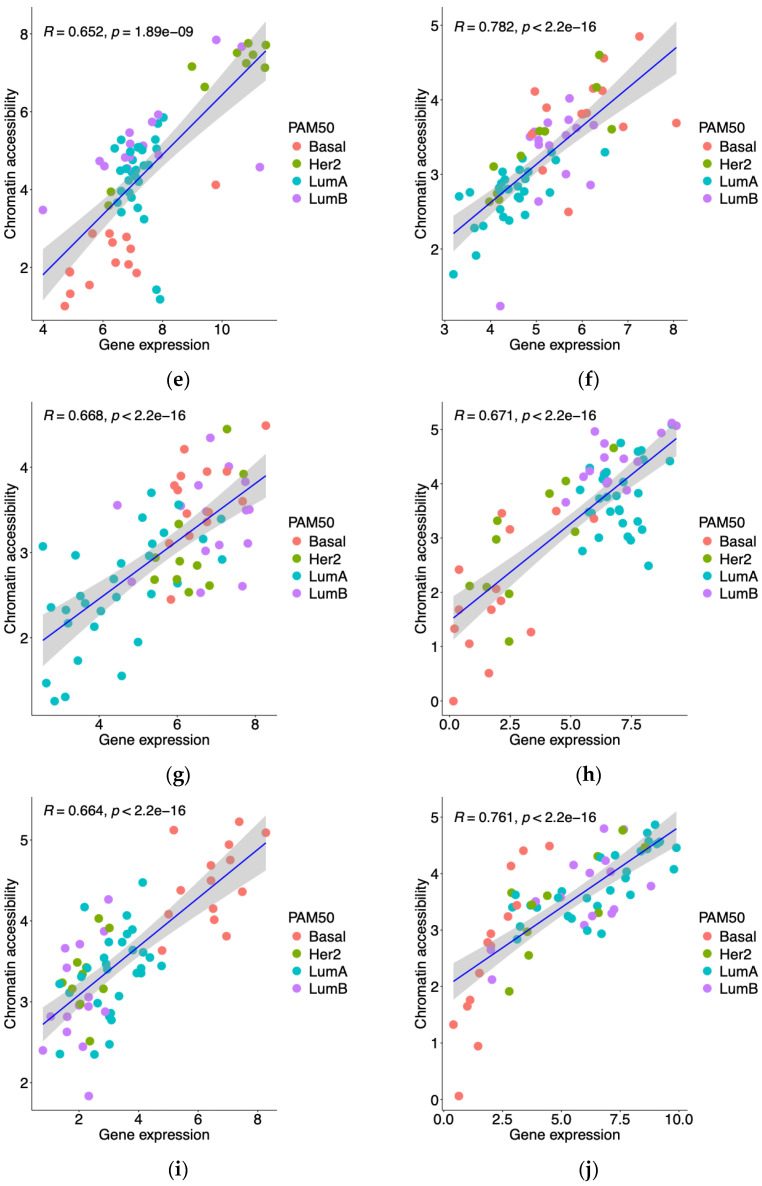
Scatter plot of gene expression and promoter accessibility for 10 PAM50 signature genes. Spearman correlation analysis was performed, considering a correlation coefficient ≥ 0.65 and a corresponding *p*-value < 0.01 as significant. Among the significantly correlated genes, 10 overlapped with the PAM50 gene signature: (**a**) *PHGDH*, (**b**) *MDM2*, (**c**) *CDH3*, (**d**) *MAPT*, (**e**) *ERBB2*, (**f**) *TYMS*, (**g**) *MYBL2*, (**h**) *ESR1*, (**i**) *FOXC1*, and (**j**) *NAT1*. Each point is color-coded according to the intrinsic subtype of breast cancer. Abbreviations: Basal, Basal-like; LumA, Luminal A; LumB, Luminal B; Her2, Her2-enriched.

To explore the relationship between chromatin accessibility at promoter regions and gene expression, we integrated RNA-seq and ATAC-seq data for 10,413 genes common to both datasets from 68 matched GDC TCGA-BRCA patients. Using Spearman correlation analysis, we identified 813 genes with the strong correlation coefficients larger than 0.65 (see Table S2 for the details). Notably, 10 of these strongly correlated genes are part of the PAM50 gene signature, which is widely used for breast cancer subtype classification: *PHGDH*, *MDM2*, *CDH3*, *MAPT*, *ERBB2*, *TYMS*, *MYBL2*, *ESR1*, *FOXC1*, and *NAT1*. Figure 1 presents scatter plots illustrating the correlation between gene expression and chromatin accessibility for these 10 genes, highlighting the relationship between changes in chromatin accessibility at promoter regions and corresponding changes in gene expression levels.

### 3.2. Recursive Feature Selection for Breast Cancer Subtype Prediction

We obtained gene expression profiles from the GEO database (GSE96058) and constructed a classification model for breast cancer intrinsic subtypes based on the 813 genes with the strong correlation coefficients between gene expression and chromatin accessibility. We divided the gene expression data into training (70%) and validation (30%) sets, followed by Z-score normalization.

Given the small sample size and the high-dimensional nature of the gene expression data, effective feature engineering was crucial. We used RFECV with SVM as the estimator, and SHAP feature importance to guide the feature selection process.

**Figure 2:**
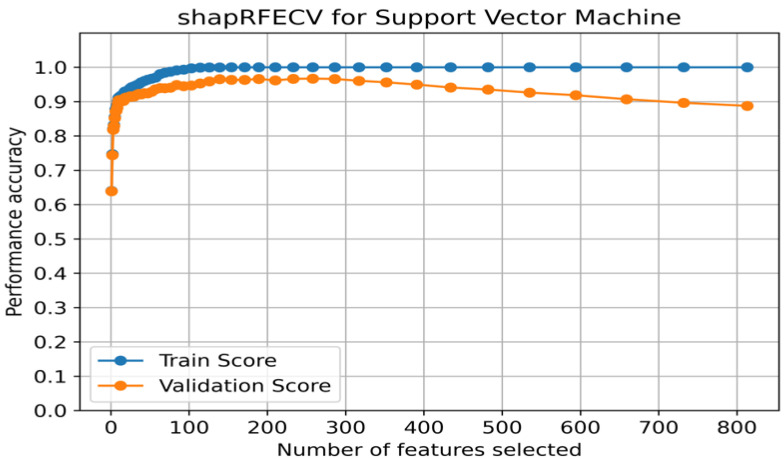
Recursive feature elimination using SHAP feature importance to find optimal genes for support vector machine. The plot shows the range of accuracy scores (from 1 to 813 genes) as a function of the number of features selected, based on 1939 training samples derived from the GSE96058 dataset.

Figure 2 illustrates the relationship between accuracy and the number of selected genes using RFECV with SVM, based on SHAP feature importance. Table S3 shows the performance of the training and validation sets for a subset of genes using the model. It is noted that the model achieved a training accuracy of 0.911 and a validation accuracy of 0.904, both exceeding 0.90, even with a set of 10 selected genes: *CDH3*, *ERBB2*, *TYMS*, *GREB1*, *OSR1*, *MYBL2*, *FAM83D*, *ESR1*, *FOXC1*, and *NAT1*. Among these 10 genes, seven overlap with the PAM50 gene signature: *CDH3*, *ERBB2*, *TYMS*, *MYBL2*, *ESR1*, *FOXC1*, and *NAT1*.

### 3.3. Prediction Performance with the 10 Selected Genes

To further evaluate classification abilities for breast cancer intrinsic subtypes using the 10 selected genes, we implemented six machine learning algorithms: SVM, LR, RF, NB, AdaBoost, and MLP. To address class imbalance and assess model performance, we applied a clustering-based under-sampling technique, which involved replacing majority class samples with centroids identified via the k-means algorithm. We then compared the performance of models built with under-sampled data to that of the base models to determine if handling class imbalance improved classification accuracy. Table S4 shows the performance of six machine learning algorithms, comparing models trained on under-sampled data with those trained on the original dataset. Overall, base models outperformed models built with under-sampled data, indicating that handling class imbalance did not improve classification accuracy. Based on these results, we did not use sampling-based methods for building our classification models. Indeed, it has been proven that the sampling-based approaches are always useful for the imbalanced datasets [35].

We conducted a grid search with stratified 10-fold cross-validation on the training data to identify the hyperparameters that yielded the highest validation accuracy scores (see Table S1 for the details). Table S5 shows the hyperparameters tested for each model, with final selection based on the highest validation accuracy scores. Table 1 presents the average performance metrics—accuracy, specificity, sensitivity, F1 score, and AUROC—from the grid search. The logistic regression model emerged as the top performer and was selected as the final model.

**Figure 3:**
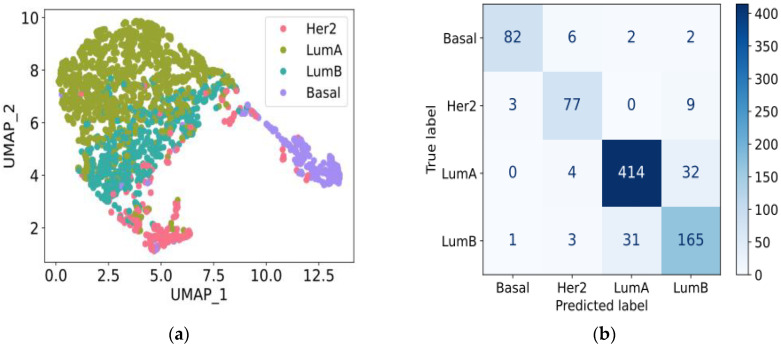
Logistic regression model training and evaluation. (**a**) UMAP plot showing the gene expression profiles of 10 selected gene from feature selection in the 1939 training samples derived from GSE96058 data. (**b**) A confusion matrix shows the consistency between the actual intrinsic subtype, and the intrinsic subtype predicted by the logistic regression model on the 831 test samples. The color axis presents the number of samples in each subtype. Abbreviations: Basal, Basal-like; LumA, Luminal A; LumB, Luminal B; Her2, Her2-enriched.

To assess its predictive capability, we evaluated the logistic regression model using unseen test data. Figure 3 shows the UMAP plot of 1939 training samples and the confusion matrix for 831 test samples. Evaluated on the test data, the logistic regression model achieved the following metrics: accuracy = 0.888, specificity = 0.956, sensitivity = 0.875, F1 score = 0.878, and AUROC = 0.981. These results underscore the model’s robustness and generalizability across diverse datasets, highlighting the critical role of the 10 selected genes in accurately classifying breast cancer subtypes and illustrating the distinct separation of intrinsic subtypes.

### 3.4. External Validation Using Other Breast Cancer Gene Expression Data

To evaluate the robustness and generalizability of our logistic regression model, we performed external validation using three independent datasets: GSE96058 (GPL18573 platform ID), GSE81538, and GSE135298. As shown in Table 2, our model demonstrated consistent performance across these validation datasets, like the results obtained from the GSE96058 test dataset (GPL1154 platform ID). This consistency highlights the model’s ability to generalize well across diverse datasets, further validating the relevance of the 10 selected genes for the subtype classification. We also reconstructed the logistic regression model using a gene expression dataset from 953 GDC TCGA-BRCA patients, which was distinct from the dataset used in the initial correlation analysis.

**Figure 4:**
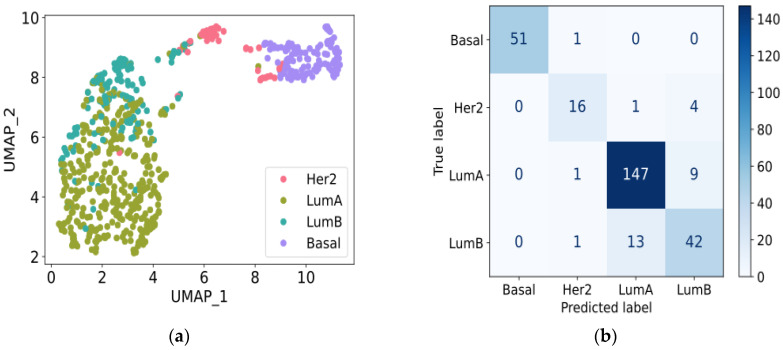
Logistic regression model training and evaluation using GDC TCGA-BRCA dataset. (**a**) UMAP plot displaying gene expression profiles of 10 selected genes in 667 training samples from GDC TCGA-BRCA data. (**b**) Confusion matrix illustrating consistency between actual and predicted intrinsic subtypes by logistic regression model on 286 GDC TCGA-BRCA test samples. Abbreviations: Basal, Basal-like; LumA, Luminal A; LumB, Luminal B; Her2, Her2-enriched.

Figure 4 shows UMAP plots for 667 training samples from the GDC TCGA-BRCA dataset and the confusion matrix for 286 test samples from the same dataset. Evaluated on the GDC TCGA-BRCA test data, the logistic regression model achieved the following metrics: accuracy = 0.895, specificity = 0.956, sensitivity = 0.857, F1 score = 0.868, and AUROC = 0.983. These results uncover the model’s robust performance and its ability to generalize across datasets again, highlighting the critical role of the 10 selected genes in accurately categorizing breast cancer subtypes and clearly illustrating the distinct separation of intrinsic subtypes.

### 3.5. Identification of Enriched Gene Sets and Motif Discovery Using the 10 Selected Genes

**Figure 5:**
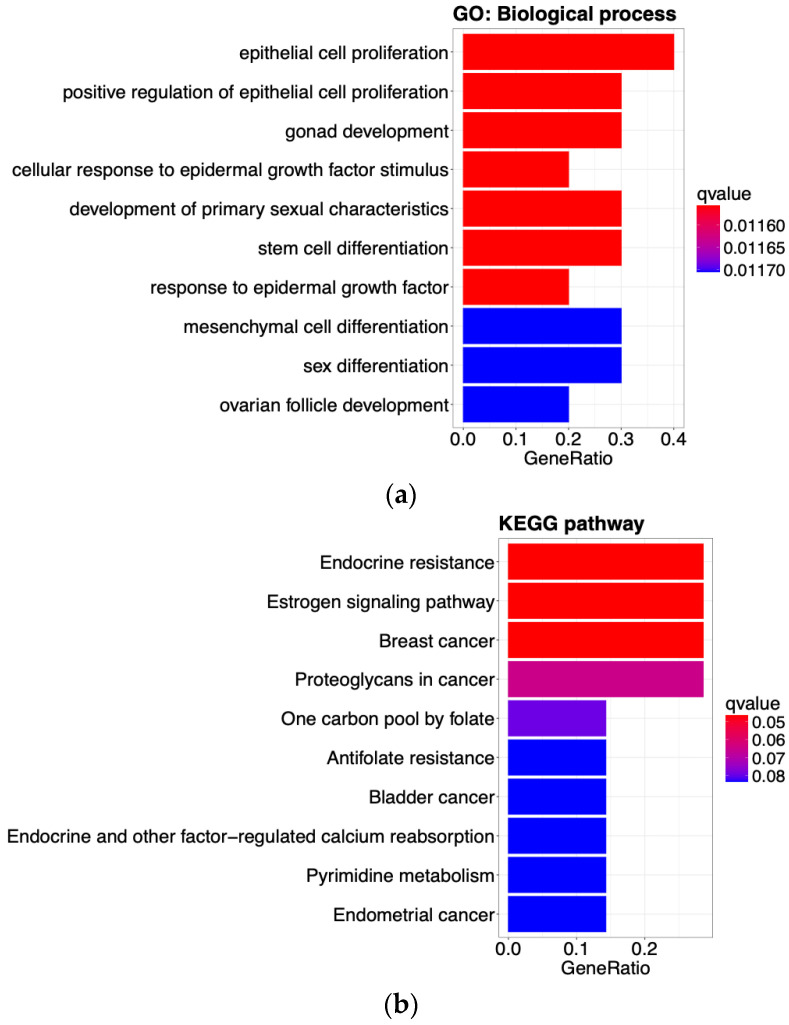
Enrichment analysis using the 10 selected genes from feature selection. (**a**) This plot shows the significantly enriched GO biological process with *q*-value < 0.05. (**b**) This plot shows the significantly enriched KEGG pathway with *q*-value < 0.1. Gene ratio is the number of overlapped genes between uploaded genes and those in the pathway category divided by the number of genes. It was sorted based on the *q*-value and expressed by color.

We conducted enrichment analyses using GO and KEGG to explore the biological functions of the 10 selected genes. In GO biological process terms, we identified 442 significantly enriched processes with a *q*-value < 0.05, including responses to glucocorticoids and estrogen, T cell differentiation, and cell proliferation (see Table S6 for the details). In KEGG pathway analysis, we identified 24 enriched pathways with a *q*-value < 0.1, including endocrine resistance, estrogen signaling pathway, prolactin signaling pathway, and breast cancer (see Table S7 for the details). Figure 5 illustrates the top 10 significantly enriched biological functions for both GO biological processes and KEGG pathways.

Next, we employed MEME-ChIP to analyze the ATAC-seq peak locations from the GDC TCGA-BRCA cohort, specifically focusing on the promoter regions of the 10 selected genes. Six known or similar motifs were identified with an E-value < 0.01: REI1 (REquired for Isotropic bud growth), EBF1 (Early B Cell Factor 1), Ebf4 (Early B Cell Factor 4), Spps (Sp1-like factor for pairing sensitive-silencing), ZNF770 (Zinc Finger Protein 770), and PATZ1 (POZ/BTB and AT Hook Containing Zinc Finger 1). Three of these motifs were excluded due to their association with Saccharomyces cerevisiae, Drosophila melanogaster, and Mus musculus.

Table 3 summarizes the remaining motifs, which are associated with the transcription factors EBF1, ZNF770, and PATZ1. These findings suggest potential regulatory mechanisms involved in the expression of these genes. Collectively, our results underscore the biological relevance of the 10 selected genes in pathways associated with hormone signaling, cancer progression, and cellular proliferation, highlighting their potential significance in breast cancer biology.

## 4. Discussion

In this study, we developed a classification model for predicting breast cancer intrinsic subtypes by integrating gene expression and chromatin accessibility profiles with machine learning algorithms. We identified 813 genes from the GDC TCGA-BRCA cohort that exhibited significant correlations between their expression levels and chromatin accessibility at promoter regions. Using RFECV with SVM as the estimator and SHAP for feature importance, we refined the gene set to 10 key genes critical for breast cancer intrinsic subtype classification.

Among these 10 genes, 7 were part of the PAM50 gene signature: *CDH3*, *ERBB2*, *TYMS*, *MYBL2*, *ESR1*, *FOXC1*, and *NAT1*. The inclusion of *GREB1*, *OSR1*, and *FAM83D*, which are not in the PAM50 gene signature, highlights the potential of our integrative approach to reveal prognostic markers related to breast cancer intrinsic subtypes. For instance, *GREB1* is a key regulatory factor of the estrogen receptor, influencing chromatin accessibility through interactions with PRC1.2, ERα, and *FOXA1*, with *FOXA1* being a notable biomarker for the luminal breast cancer subtype [36,37,38]. Amir et al. demonstrated that *OSR1* is significantly down-regulated across Luminal A, Luminal B, HER2-positive, and TNBC (triple-negative breast cancer) subtypes and interacts with hsa-miR-21-5p, a microRNA biomarker for breast cancer diagnosis identified by Min Liu et al. [39,40]. Additionally, *FAM83D* has been identified as an independent biomarker for breast cancer intrinsic subtypes through meta-analysis, and separate research has shown that it is associated with immune-infiltrative subtypes while displaying variable correlations with stromal cell infiltration and tumor stem cells [41,42,43].

We evaluated the model’s performance using metrics including accuracy, specificity, sensitivity, F1 score, and AUROC, demonstrating its effectiveness in subtype classification. Validation on independent datasets confirmed the model’s robustness and underscored the relevance of the 10 genes across different datasets. Additionally, retraining the model on GDC TCGA-BRCA data, which were not initially used, further validated its generalizability and strong performance in subtype classification. Previously, Okimoto et al. demonstrated that a support vector machine classifier based on 36 subset genes from the PAM50 gene signature achieved accuracy comparable to models using the full PAM50 gene signature [3]. Our findings highlight that the 10 genes selected through our integrative analysis are highly effective for classifying breast cancer intrinsic subtypes, even though not all are part of the traditional PAM50 gene signature.

Additionally, we conducted enrichment analysis and motif discovery to explore the biological functions and pathways associated with these genes. This analysis revealed significant connections with immune responses, hormone signaling, cancer progression, and cellular proliferation, underscoring their relevance in breast cancer biology. Motif discovery identified motifs associated with three transcription factors—EBF1, ZNF770, and PATZ1—suggesting potential regulatory mechanisms influencing gene expression and breast cancer intrinsic subtypes. For example, the EBF1 motif is a crucial regulator of subtype-specific methylation and gene expression, with notably high expression in triple-negative breast cancer [44,45]. Several studies have shown that *ZNF770* is significantly upregulated across Luminal A, Luminal B, HER2-positive, and TNBC subtypes and interacts with miR-3656, with expression changes observed following HER2-targeted drug treatment, as identified by Lisa et al. [39,46]. Meanwhile, Fangchao et al. found that *CBLL1* expression was significantly higher in Luminal A and Luminal B subtypes compared to others, and that *ZNF770* expression was significantly lower in the CBLL1-low group compared to the CBLL1-high group within the luminal subtype [47]. Furthermore, *PATZ1* is targeted by miR-29b, a microRNA biomarker for the HER2-enriched subtype, with its expression negatively associated with this subtype, and overexpression of miR-29b inhibits breast cancer cell proliferation and induces apoptosis primarily by downregulating STAT3 protein levels [48,49].

These findings highlight the potential of the 10 informative genes as promising biomarkers. However, several limitations should be acknowledged. Firstly, due to computational constraints, it was impractical to exhaustively test all possible parameter values for machine learning algorithms; thus, we relied on grid search with stratified 10-fold cross-validation and preselected hyperparameter values. Secondly, the ATAC-seq data from the GDC TCGA-BRCA cohort had a limited sample size, which may impact result robustness. To mitigate this, we performed Spearman correlation analysis at the individual sample level to link open chromatin with gene expression, and validated our model across multiple independent gene expression test sets. Finally, since our study was based solely on bioinformatics analyses, experimental validation is necessary to confirm the reliability of these genes as biomarkers for accurately predicting breast cancer intrinsic subtypes.

Furthermore, recent advances have highlighted the importance of multiomics integration, particularly in the classification of breast cancer subtypes using machine learning algorithms [50,51,52,53]. Lin et al. developed DeepMO, which classifies breast cancer subtypes based on gene expression, DNA methylation, and copy number variation [54]. Choi et al. proposed moBRCA-net, which employs an attention-based neural network to classify breast cancer subtypes based on gene expression, DNA methylation, and miRNA expression [55]. Huang et al. developed a differential sparse canonical correlation analysis network (DSCNN) model, a multitask deep learning neural network based on gene expression and DNA methylation [56]. Looking ahead, future research could benefit from integrating chromatin accessibility data with other multiomics profiling datasets. This integration has the potential to enhance our understanding of the complex biological mechanisms underlying breast cancer, potentially revealing novel insights into gene regulation and epigenetic modifications specific to various breast cancer subtypes. Moreover, it is necessary to develop more advanced machine learning algorithms capable of effectively integrating multimodal omics data and systematically interpreting biological phenomena. In addition, exploring temporal dynamics in multiomics data to capture the evolving nature of cancer progression, advancing single-cell multiomics integration techniques to better understand tumor heterogeneity, and focusing on translating multiomics findings into clinically actionable insights are also required.

## 5. Conclusions

In conclusion, we identified 10 signature genes for breast cancer intrinsic subtypes by integrating gene expression and chromatin accessibility data with machine learning algorithms. These genes are primarily associated with immune response, hormone signaling, cancer progression, and cellular proliferation. Their use can help reduce costs and complexity in both research and clinical settings. This approach not only improves breast cancer subtype classification but also holds potential for applications beyond breast cancer research. Our findings contribute to biomarker identification and advance our understanding of cancer biology, supporting progress in precision oncology. Future work should focus on incorporating chromatin accessibility data with other omics profiles and developing advanced machine learning algorithms for multimodal data integration, and exploring temporal and single-cell multiomics approaches. These efforts will not only enhance our understanding of breast cancer biology but also improve the accuracy and clinical utility of subtype classification methods, potentially leading to more personalized treatment strategies.

## Figures and Tables

**Table 1:** Prediction performance using grid search with stratified 10-fold cross-validation. Abbreviations: SVM; support vector machine, LR; logistic regression, RF; random forest, NB; naïve Bayes, AdaBoost; adaptive boosting based on decision trees, MLP; multilayer perceptron.

Models	Accuracy	Specificity	Sensitivity	F1 Score	AUROC
SVM	0.904	0.960	0.888	0.894	0.983
LR	0.905	0.961	0.889	0.895	0.984
RF	0.862	0.939	0.824	0.846	0.970
NB	0.858	0.941	0.833	0.842	0.969
AdaBoost	0.843	0.935	0.819	0.831	0.937
MLP	0.896	0.957	0.874	0.882	0.981

**Table 2:** The performance of the logistic regression models with three external datasets.

Models	Accuracy	Specificity	Sensitivity	F1 Score	AUROC
GSE96058 (Illumina Nextseq 500)	0.901	0.957	0.856	0.872	0.983
GSE81538	0.888	0.960	0.904	0.896	0.985
GSE135298	0.878	0.955	0.845	0.824	0.991
Average	0.889	0.957	0.868	0.864	0.986

**Table 3:** Sequence logos of the DNA motifs determined by MEME-ChIP analysis enriched by the selected 10 genes (E-value < 0.01).

Motifs	E-Value	Known or Similar Motifs
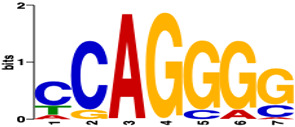	6.0 × 10^−05^	EBF1
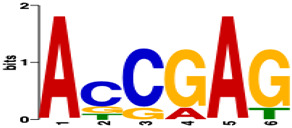	1.5 × 10^−03^	-
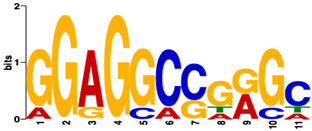	5.4 × 10^−03^	ZNF770/PATZ1
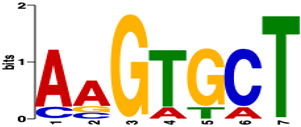	9.9 × 10^−03^	-

## Data Availability

RNA-seq and ATAC-seq data for integration analysis have been deposited in the Xena Genome Browser (https://xenabrowser.net/, accessed on 15 August 2024) and are publicly available as of the date of publication. RNA-seq data used for model construction and evaluation have been deposited in the NCBI Gene Expression Omnibus (https://www.ncbi.nlm.nih.gov/geo/, accessed on 15 August 2024) and are also publicly available as of the date of publication.

## Supplementary Materials

The following supporting information can be downloaded at: https://www.mdpi.com/article/10.3390/biology13100799/s1, Figure S1: The distribution of 215,920 GDC TCGA-BRCA specific peaks; Table S1: Hyperparameters used for model tuning using a grid search with stratified 10-fold cross-validation; Table S2: Significant correlation coefficient between gene expression and chromatin accessibility at the promoter region; Table S3: The result of recursive feature elimination based on SHAP feature importance; Table S4: Model performance on the original and balanced under-sampled data, evaluated using stratified 10-fold cross-validation repeated 10 times (mean ± std); Table S5: Finally selected hyperparameters by a grid search with stratified 10-fold cross-validation; Table S6: GO Biological Processes enriched for the selected 10 genes; Table S7: KEGG pathways enriched for the selected 10 genes. This manuscript is accompanied by a GitHub repository (https://github.com/jeong2624/ML_BRCA-subtype/tree/main, accessed on 15 August 2024) that contains the necessary codes and scripts for this analysis.
