# Supplementary material for: Integrative Analysis of ATAC-Seq and RNA-Seq through Machine Learning Identifies 10 Signature Genes for Breast Cancer Intrinsic Subtypes
Source: Biology (Basel). 2024 Oct 7;13(10):799. doi: 10.3390/biology13100799 (PMC11505269; doi:10.3390/biology13100799)
Supplement: Supplementary file 1 [file biology-13-00799-s001.zip › Supplementary_Figure.pdf]

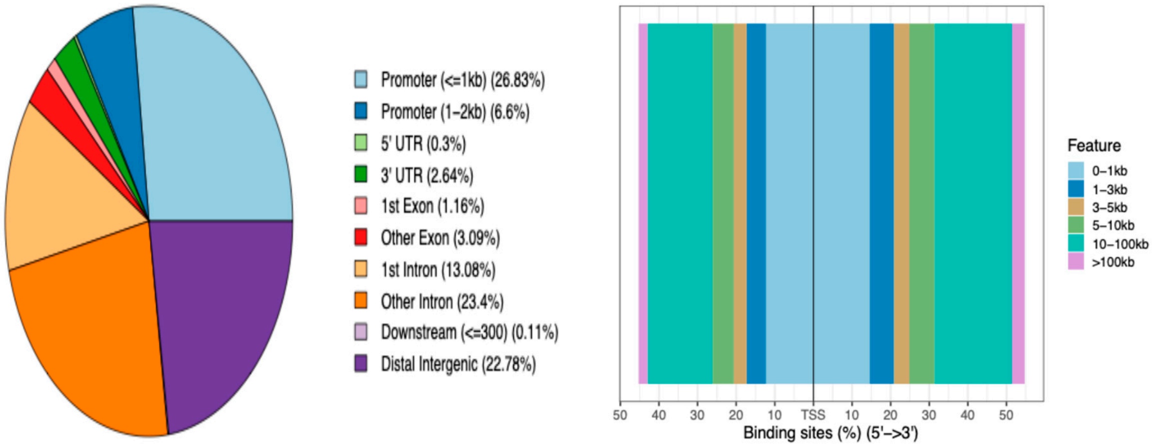


(**a**) (**b**)

**Figure S1.** The distribution of 215,920 GDC TCGA-BRCA specific peaks. Regions within 2 kb upstream and downstream of the TSS were defined as promoter regions, while all others were classified as distal regions. (**a**) Pie chart showing the genomic distribution peaks in each functional region of genes identified in ATAC-seq data. (**b**) Genomic distribution of peak binding sites relative to the TSS of the nearest genes.
